# Advanced Glycation End-Products Reduce Collagen Molecular Sliding to Affect Collagen Fibril Damage Mechanisms but Not Stiffness

**DOI:** 10.1371/journal.pone.0110948

**Published:** 2014-11-03

**Authors:** Gion Fessel, Yufei Li, Vincent Diederich, Manuel Guizar-Sicairos, Philipp Schneider, David R. Sell, Vincent M. Monnier, Jess G. Snedeker

**Affiliations:** 1 Department of Orthopedics, Balgrist University Hospital, University of Zurich, Zurich, Switzerland; 2 Institute for Biomechanics, ETH Zurich, Zurich, Switzerland; 3 Institute for Chemical and Bioengineering, ETH Zurich, Zurich, Switzerland; 4 Paul Scherrer Institute, Villigen, Switzerland; 5 Faculty of Engineering and the Environment, University of Southampton, Southampton, United Kingdom; 6 Department of Pathology, Case Western Reserve University, Cleveland, Ohio, United States of America; 7 Department of Biochemistry, Case Western Reserve University, Cleveland, Ohio, United States of America; Queen Mary University of London, United Kingdom

## Abstract

Advanced glycation end-products (AGE) contribute to age-related connective tissue damage and functional deficit. The documented association between AGE formation on collagens and the correlated progressive stiffening of tissues has widely been presumed causative, despite the lack of mechanistic understanding. The present study investigates precisely how AGEs affect mechanical function of the collagen fibril – the supramolecular functional load-bearing unit within most tissues. We employed synchrotron small-angle X-ray scattering (SAXS) and carefully controlled mechanical testing after introducing AGEs in explants of rat-tail tendon using the metabolite methylglyoxal (MGO). Mass spectrometry and collagen fluorescence verified substantial formation of AGEs by the treatment. Associated mechanical changes of the tissue (increased stiffness and failure strength, decreased stress relaxation) were consistent with reports from the literature. SAXS analysis revealed clear changes in molecular deformation within MGO treated fibrils. Underlying the associated increase in tissue strength, we infer from the data that MGO modified collagen fibrils supported higher loads to failure by maintaining an intact quarter-staggered conformation to nearly twice the level of fibril strain in controls. This apparent increase in fibril failure resistance was characterized by reduced side-by-side sliding of collagen molecules within fibrils, reflecting lateral molecular interconnectivity by AGEs. Surprisingly, no change in maximum fibril modulus (2.5 GPa) accompanied the changes in fibril failure behavior, strongly contradicting the widespread assumption that tissue stiffening in ageing and diabetes is directly related to AGE increased fibril stiffness. We conclude that AGEs can alter physiologically relevant failure behavior of collagen fibrils, but that tissue level changes in stiffness likely occur at higher levels of tissue architecture.

## Introduction

Aging is associated with a progressive stiffening of connective tissues of the body. These include hard connective tissues of the skeleton [1], [2] and soft tissues of the cardiovascular system [3], lungs [4], skin [5] and tendons [6]. The stiffening of tissue cannot be explained by increased collagen content alone [3], [6] and is accompanied by age-associated yellowing of the collagen matrix, decreased collagen solubility, and heightened collagen resistance to protease breakdown. Certain metabolic diseases, particularly diabetes, accelerate these phenomena that have been causally linked to non-enzymatic glycation of proteins [7]–[10]. Protein glycation ultimately leads to the formation of advanced glycation end-products (AGEs), with inter- and intra-molecular AGE cross-links suspected to affect molecular and cellular function, with according functional consequences at the tissue level. The biochemical reactions involved in AGE formation are well documented, as are AGE stimulated cellular production of reactive oxygen species, and the activation of inflammatory signaling cascades via AGE signaling receptors (RAGEs) [11]. Together with changes in tissue elasticity and associated changes in the mechanical stimuli that drive resident cell behavior, these factors may play a crucial role in loss of tissue homeostasis and progression of connective tissue disease.

AGEs are formed when endogenous carbonyl groups of reducing sugars non-enzymatically react with free amino groups of proteins. Although glucose is relatively slow in reacting with most proteins, highly reactive dicarbonyl compounds are generated upon glucose auto-oxidation. Dicarbonyls can also form as metabolic by-products of glycolysis including the products glyoxal, methylglyoxal (MGO) and 3-deoxyglucosone, all of which can interact with extracellular proteins to rapidly form AGEs [12]. So far, there is no direct experimental evidence linking AGEs with increases in collagen fibril stiffness, which in turn would cause increased stiffness at higher levels of tissue architecture. Although the mechanical effects of AGEs at the molecular and supramolecular levels are poorly understood, this link seems plausible and has been widely presumed to exist on the basis of the well documented correlation between AGE markers (pentosidine; auto-fluorescence) and increasing tissue stiffness [13].

**Figure 1:**
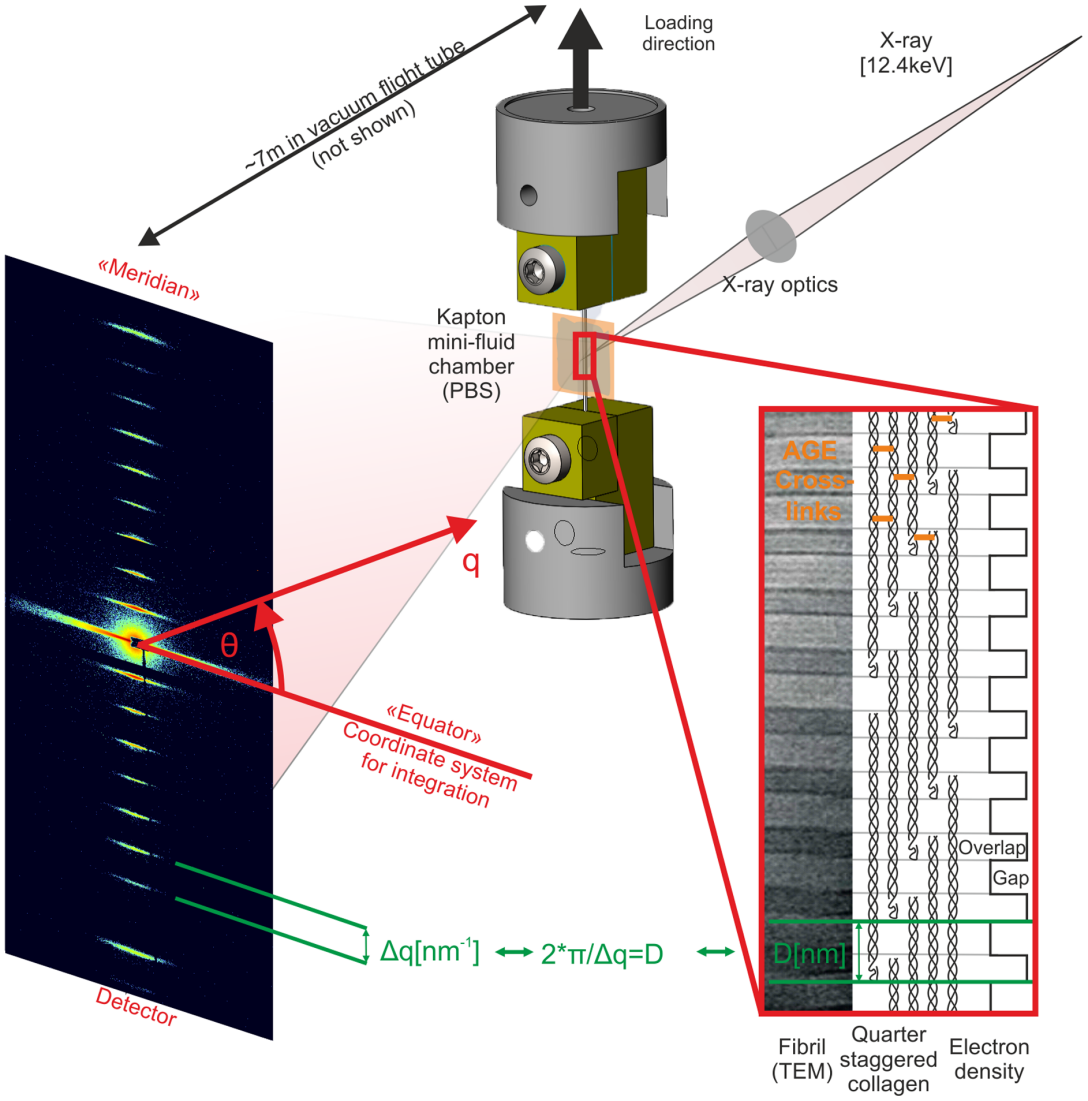
Schematic of experimental principles. **Left)** 2D diffraction patterns from tendons are dominated by the meridional Bragg reflections corresponding to ∼67.5 nm and harmonic frequencies of this spacing. **Right)** This pattern results from the quarter-stagger model of collagen. More simply, this can be seen as “gap” and “overlap” regions with high and low electron dense areas that scatter X-rays as wide periodic slits [63]. **Middle)** The meridional intensities were integrated over azimuthal (θ) sectors, which resulted in 1-D intensity profiles, I [a.u.] vs. q [nm^−1^]. The peak positions or correspondingly the distance between peaks) were used to calculate the length of the periodicity (Bragg's law) during tensile experiments. Changes of the peak spacing served then as averaged measure for collagen fibril elongation: absolute deformation (D) and relative fibril strain).

**Figure 2:**
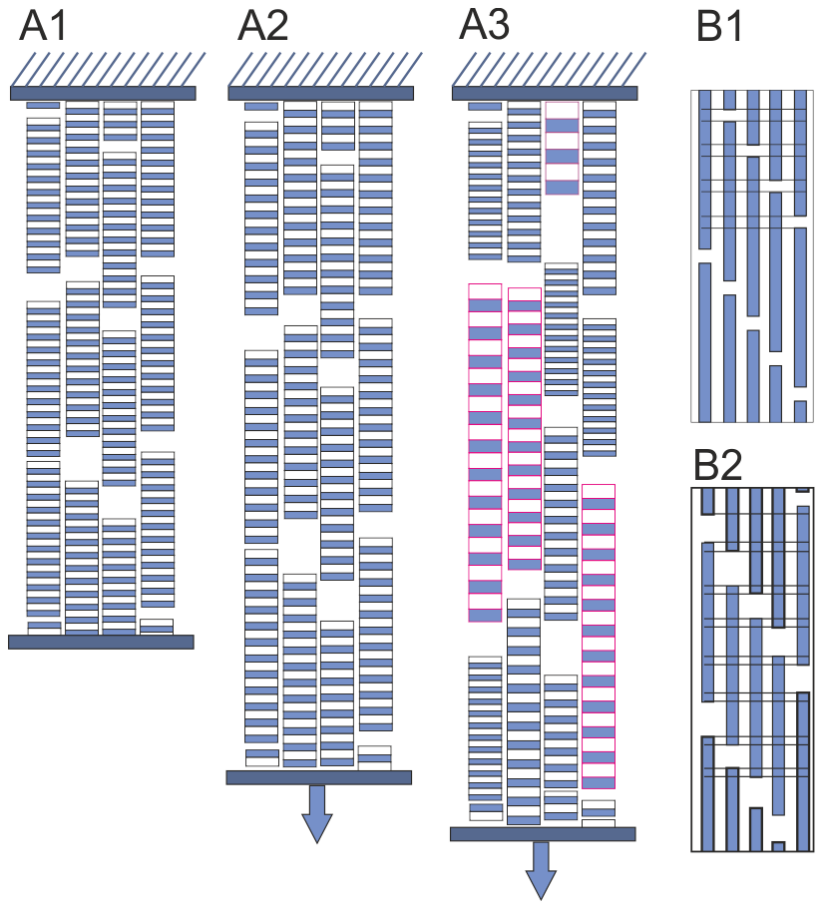
Sketch of collagen fibril deformation and failure. **A1)** Unloaded collagen fibrils. **A2)** With applied strain (blue arrows), the collagen fibril D-periods increase homogenously and are approximately equally long. This results in sharp peaks of the meridional collagen reflections. **A3)** Post yielding, the collagen fibrils are deformed more heterogeneously. This resulted in the broader peaks, measured as FWHM. Some AGE samples this heterogeneity resulted in two distinct populations of fibrils, which resulted in peak splitting. **B1)** Individual collagen fibril from A1 composed of collagen molecules with shown D-periodic pattern. **B2)** Apart from the molecule elongation the fibril elongation consists of side-by-side sliding of collagen molecules, which results in a decreased overlap (O) region, while the D-period increases (not shown). This decreases the O/D ratio.

Collagen molecules are composed of triple helical amino acid chains that form a molecular unit with an approximate diameter of 1.5 nm and length of 300 nm. These molecules aggregate into collagen fibrils (functional supramolecular structures with diameters of 10–500 nm) adopting a so-called “quarter-stagger” model that yields the characteristic fibril striation that can be seen in electron micrographs (Fig. 1). This pattern is due to the length of each collagen molecule being 4.4 times that of the periodic striation (D
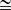
67 nm). The consequence of this geometric configuration is a gap of approximately 0.54 D length between the ends of successive collagen molecules. These nano-scale distances can be measured by small-angle X-ray scattering (SAXS) to assess molecular deformations during mechanical loading [14]–[16]. Past measurements have employed SAXS to estimate collagen molecule [17] and fibril stiffness [18]. Measurements of the deformations underlying collagen fibril elongation are generally considered to reflect the cumulative effects of molecular elongation (i.e. “strain”) and side-by-side sliding of molecules [15], [18] (Fig. 2). Experiments and models have shown that both of these phenomena are strongly dependent upon enzymatic lysyl oxidase (LOX) cross-links between neighboring collagen molecules [19]–[21].

In contrast to the enzymatic cross-links involved in tissue maturation, very little evidence exists regarding the molecular-level mechanical effects of stochastically distributed non-enzymatic cross-links (AGEs) in collagen. While an atomistic model was recently used to predict preferential sites of AGE cross-links [22], the role of AGEs has been mostly explored at the tissue level. For instance, increasing AGE content has been correlated to reduced bone toughness and increased fracture risk, a relationship that has been demonstrated experimentally [23], [24] and through numerical modeling [25].

The goal of the present investigation was to more directly quantify the mechanical consequences of AGEs on collagen fibril failure behavior and viscoelasticity. This study employed synchrotron small-angle X-ray scattering and mechanical testing to reveal how collagen fibril deformations are altered by MGO induced AGEs in rat tail tendon fascicles. The rat tail tendon fascicle is a widely employed experimental model to study collagen structure-function, and was previously employed by us to characterize the effects of AGEs on collagen fiber kinematics at the cellular scale (∼10 spatial resolution) using multiphoton confocal microscopy [26]. In this earlier work, we observed drastically diminished tendon viscoelasticity in MGO treated tendons and a corresponding loss of collagen fiber sliding. The mechanisms behind this potentially critical functional loss remained unclear. The intent of the present study was to focus on the underlying molecular effects of AGEs, hoping to gain mechanistic insight into these functional deficits that may underlie loss of tissue homeostasis and play a central role in tissue disease.

## Results

### Tissue mechanical effects of MGO

**Figure 3:**
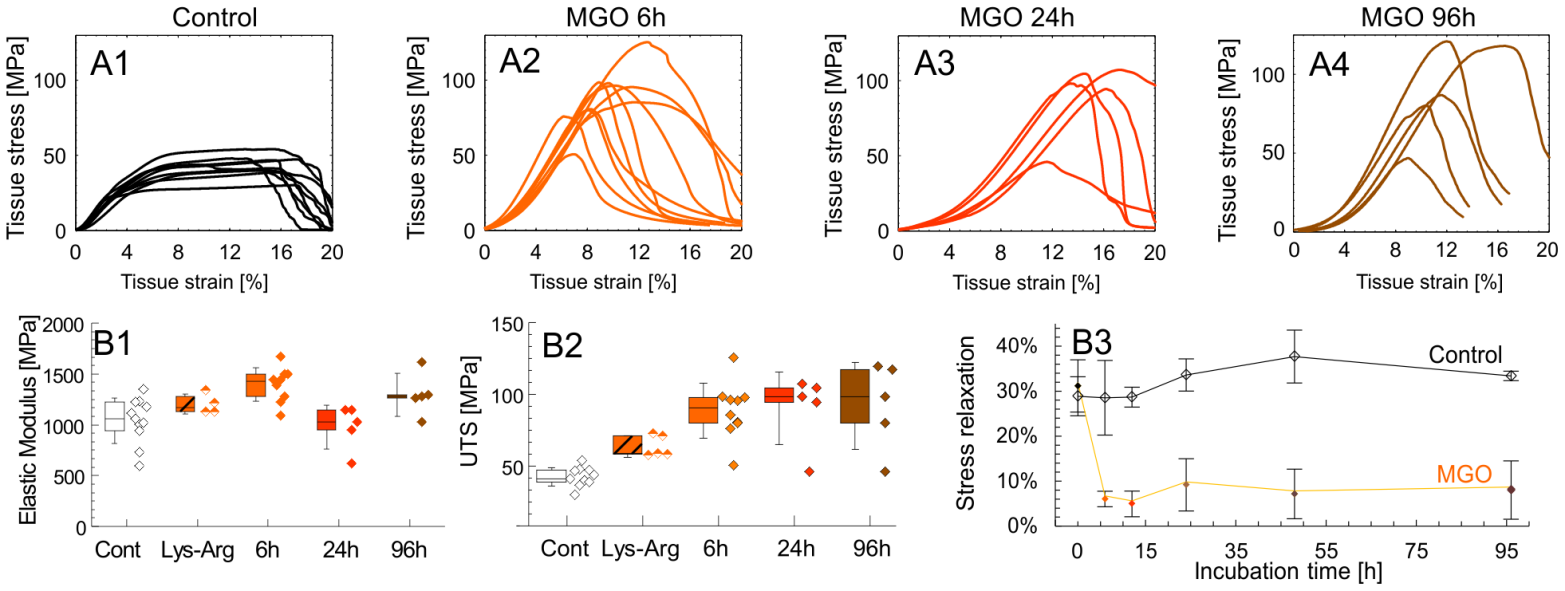
Mechanical characterization of the tendon-AGE model. **Row A:** Stress-strain from the first experimental set. Except for the MGO 96 h group, these curves were measured simultaneously with the SAXS experiments. **Row B:** A selection of material properties extracted from row A: **B1)** Elastic modulus was obtained in the linear range. Given are: mean (horizontal bar), percentile box (25% and 75%) and ±1 SD. The specificity of the MGO treatment was shown by the addition of L-lysine and L-arginine (LYS-ARG group) to the MGO solution and compared to MGO controls. For ease of reading this control is not shown. **B2)** Ultimate tensile strength (UTS). **B3)** Separate stress relaxation experiment (n = 5) was performed with repeated measurements on the same samples and compared to a corresponding control (n = 5). Stress relaxation is reported as: **σ**_R_(175 s) = 1-**σ**(175 s)/**σ**(0 s) [%].

Consistent with our previous study [26], tissue stiffness and stress relaxation were heavily affected in rat tail tendon fascicles treated with highly purified MGO to induce formation of AGEs [27] *(for details on MGO synthesis and quality control see: Figure S1)*. Dissected fascicles were incubated for 6 h, 24 h or 96 h in either 30 mM MGO solution or in buffer only. Subsequent quasi-static mechanical tests revealed that mechanical properties were most changed after the first 6 h of treatment, with only incremental additional effects afterward (analysis of variance (ANOVA): p<0.001) (Fig. 3). We observed a significant increase of elastic modulus by 35% in the linear range of the stress-strain curve (post hoc: p = 0.03) (Fig.: 3 B1). Ultimate tensile strength increased by 110% after 6 h of treatment (post hoc: p = 0.03) with no significant further increase with prolonged incubation times (Fig.: 3 B2). Tissue relaxation was significantly reduced by the 6 h treatment, a reduction that also plateaued at later time points (repeated measures (RM) ANOVA, treatment time: p<0.001, treatment group: p = 0.05) (Fig. 3 B3). We verified that the observed mechanical effects were actually evoked by MGO reaction with lysine and arginine [9], [12], [28], as confirmed by effectively inhibiting the mechanical changes upon addition of 20 mM L-lysine and 20 mM L-arginine to the MGO cross-linking solution (Fig. 3 B1 and B2).

### Collagen fibril deformation under load – supramolecular failure behavior

Collagen fibril failure behavior (as inferred from tissue level failure behavior and corresponding measures of fibril level strain) was heavily affected by MGO treatment with higher peak fibril strain at tissue failure. The apparent fibril failure mode also changed upon MGO treatment, with a more abrupt (“brittle”) onset of collagen fibril disruption.

**Figure 4:**
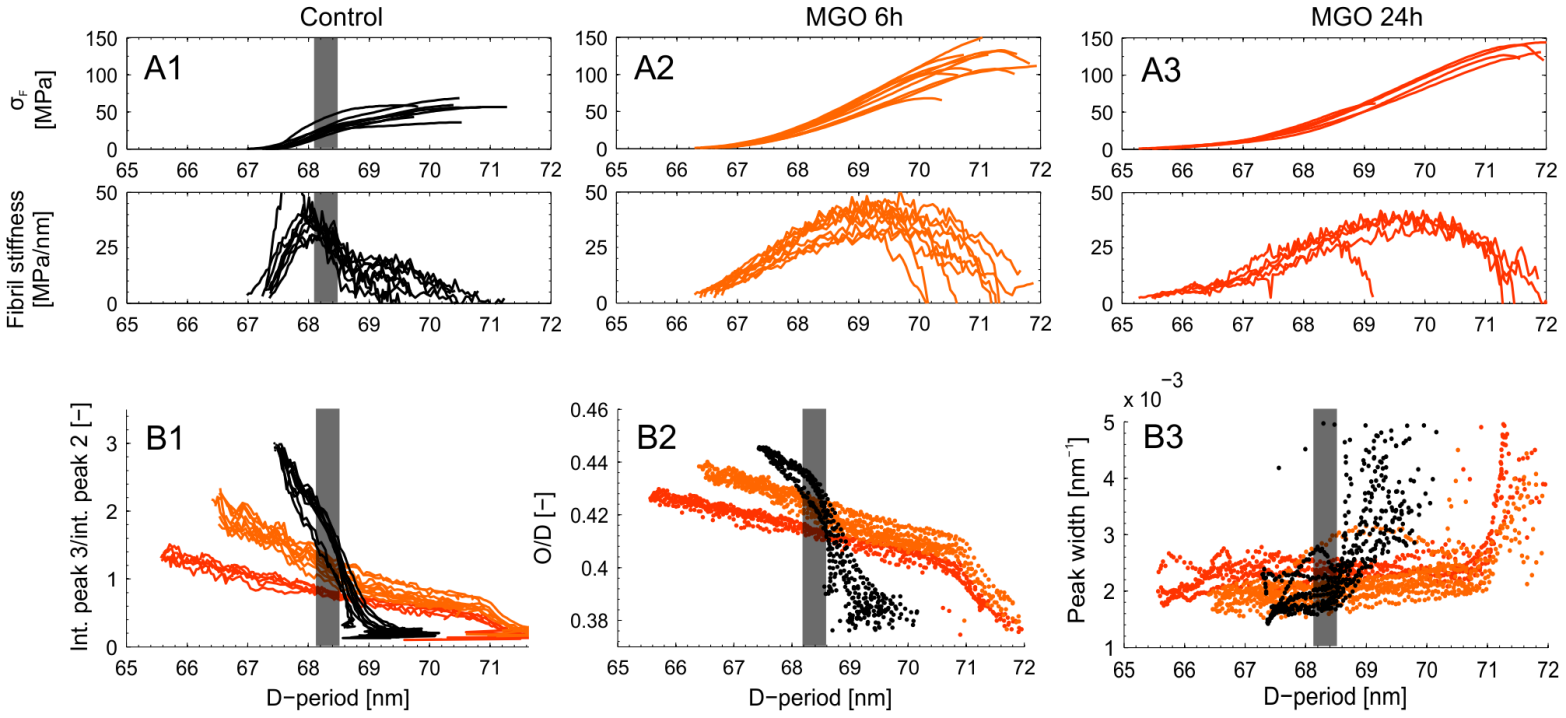
Results from SAXS experiments. **Row A)** Collagen fibril stress) vs. D-period. Normalized collagen fibril stress was calculated using the rule of mixtures. The numerical gradient (slope) of the D-period vs. collagen fibril stress curves was used to measure fibril stiffness. Peak stiffness was used as the parameter for statistical interference testing. The second experimental set (w/o a 24 h MGO group) is not plotted here since over all the stresses were approximately one third lower but with equal group effects (two-way ANOVA: interactions term, p = 0.531). **B1)** Ratio of the integrated intensities for the 2^nd^ and 3^rd^ order meridional collagen reflections. **B2)** Relative contribution of overlap (O) region to the D-period (O/D) vs. D-period, when assuming a two-phasic approximation of the D-period electron density [17], [63]. **B3)** Peak widths (FWHM) from fitted Gaussians measured during the tensile experiments vs. D-period length. **Note:** The grayed area in the insets represents the range of yield point of the control samples as reference.

**Figure 5:**
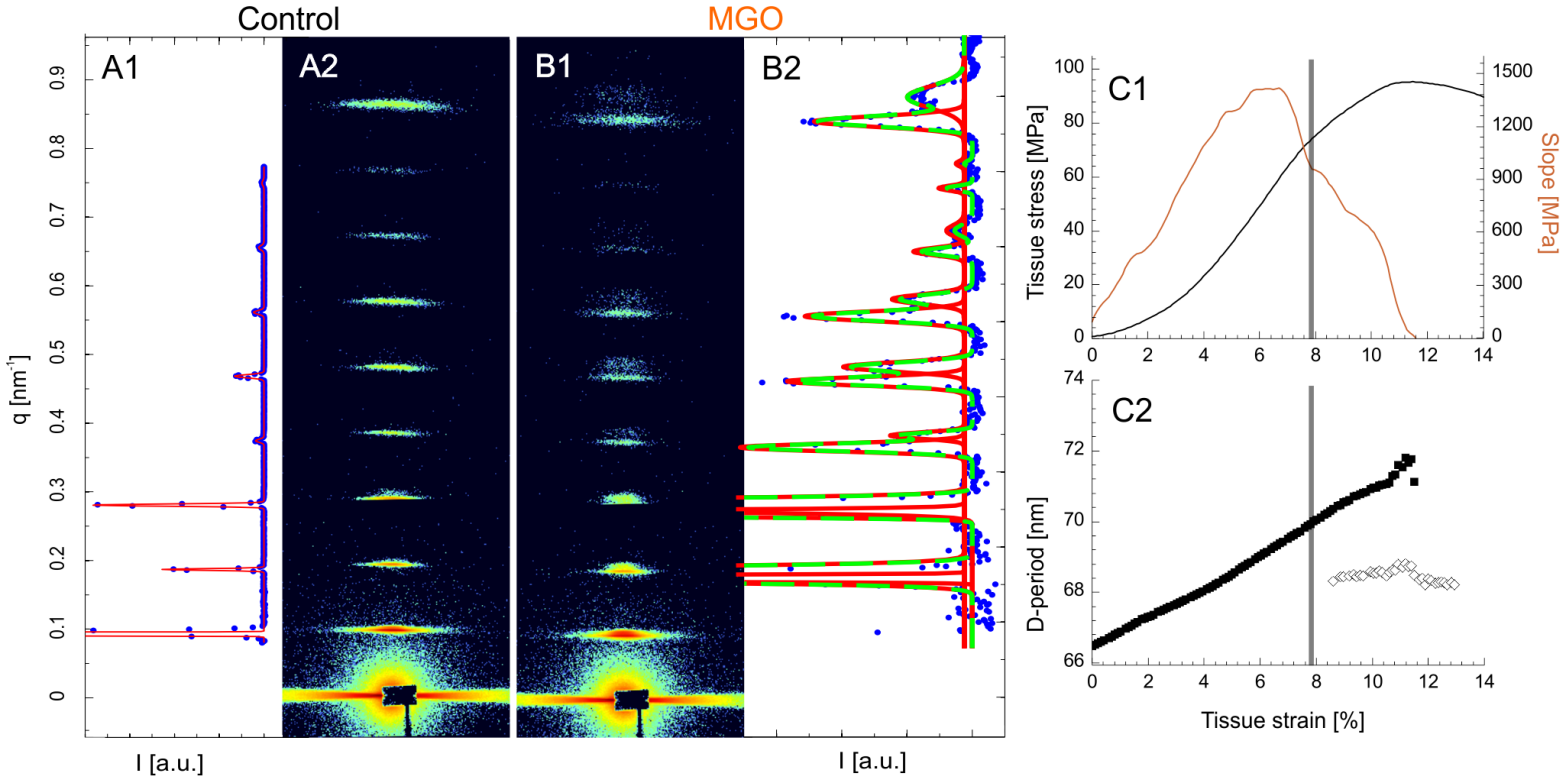
Failure mode of collagen fibrils. **A+B)** Diffraction patterns recorded after yielding. **A)** A control sample. **B)** A 6 h MGO sample. The corresponding 1D azimuthal averages are indicated by blue dots. The intensity range is scaled to show the full scale from the 3^rd^ diffraction peak. Peaks were fitted to Gaussian functions (red). In some MGO samples the best fit was bimodal Gaussian (green) in account of the peak splitting as the sum of two Gaussians (red). The first peak is not shown. **C)** Stress-strain curve of a MGO sample that showed peak splitting shortly after yielding (grey horizontal bar). The gradient of the stress-strain curve, which is a local estimate of elastic modulus, is also shown. D-period estimates of the same sample is also shown (3^rd^ reflection), with the loaded and deformed collagen fibrils (♦) and the more relaxed ones (◊).

Collagen fibril deformation during tendon tensile loading was measured at the coherent small-angle X-ray scattering beamline at the Swiss Light Source, Paul Scherrer Institute, Switzerland. Two experimental sets were measured, set 1: control (n = 9), 6 h MGO (n = 10), 24 h MGO (n = 5) and set 2: control (n = 5), 6 h MGO (n = 5). Collagen fibril deformation was measured as absolute and relative increase of D-periodic length. Fibril deformation and fibril strain was a highly linear function of tissue strain, although the force-deformation response of collagenous connective tissues is typically non-linear (Fig. 4 A and 5 C). In all groups, the meridional collagen scattering peak width, measured as FWHM, remained approximately constant up to yield point (Fig. 4 B3). This indicates homogeneously distributed fibril strains [29] (Fig. 2). After yielding a sudden broadening of the peak occurred, indicating a more heterogeneous collagen fibril strain distribution that reflects the onset of non-elastic (plastic) deformation and damage accumulation with increasing numbers of ruptured fibrils and disrupted inter-fibril matrix.

### Collagen fibril stiffness – analysis of molecular strain and sliding

Analysis of the molecular deformations within individual fibrils revealed that sliding between neighboring collagen molecules was heavily affected by MGO treatment, but that overall stiffness of the fibrils was not altered.

To analyze the molecular mechanisms behind these observed fibril level changes, we decomposed fibril elongation into homogenous, affine molecule elongation (i.e. strain) and molecular side-by-side sliding. The effect of molecular sliding was indicated as a relative length change of gap and overlap regions (Fig. 2) [18]. Such changes in the ratio of gap to overlap and consequently the overlap to D-period (O/D), can be observed as a change in relative intensity (I of order n) of the meridional D-period reflections as described by 
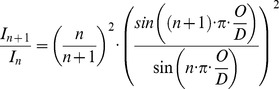
when a simple two-phase arrangement is assumed with lower and higher electron dense regions representing the gap and overlap in the collagen fibril (Fig. 1 and 2 B) [18]. This assumption compares well to more sophisticated models of axial collagen molecular conformation [14], [15], [30]. The ratio of intensities of the 2^nd^ and 3^rd^ order reflections were analyzed to assess changes in length of the overlap region (O), with this ratio decreasing monotonically with increasing fibril strain (Fig. 4 B1, Fig. 4 B2). This relative shortening of the overlap region was less pronounced after MGO treatment, and the onset of overlap shortening was delayed until higher tissue strains. However, MGO treatment was also accompanied by a shortening of the overlap region and the D-period at pre-load.

Furthermore, 7 samples out of 15 in the 6 h MGO group showed diffraction patterns with onset of peak splitting near mechanical yielding of the tissue (Fig. 5). The splitting implies onset of a bimodal distribution of collagen fibrils, with one subpopulation representing mechanically loaded (and stretched) fibrils and the other subpopulation in the distribution reflecting fibrils in the processes of relaxing toward their unstressed length [31]. In control tendons the diffraction pattern could not be decomposed to the sum of two different Bragg reflections, indicating a characteristically less abrupt mode of fibril damage, characterized by a respective loss of the quarter-staggered molecular arrangement.

The D-period length measured at a given level of applied pre-stress (D_0_) was shorter in accordance with duration of the MGO treatment (control: 67.7±0.04 nm; 6 h MGO: 66.86±0.17 nm; 24 h MGO: 65.91±0.07 nm; two-way ANOVA, treatment group and time of treatment: p<0.001, all post hoc: p<0.001). For this reason we relied on collagen fibril stiffness, a measurement based on fibril deformation D, to discriminate the mechanical effects of the treatment between groups and rather than relying on collagen fibril modulus, which is based on fibril strain. This ensured that the measurement for fibril elongation was not biased by the different D_0_ length between groups.

Additional experiments were performed to estimate potential biases related to cross-linking of the non-collagenous matrix (i.e. cells, proteoglycans, elastin) and potential swelling artifacts (*see: Non-collagenous matrix characterization in Text S1*). After characterizing the mechanical properties of the non-collagenous matrix, we used the rule of mixtures: **σ**_T_ = (1 - f) * **σ**_M_+f * **σ**_F_ [32], [33], with an assumed collagen fibril area fraction of f = 74.4%±4.7% for all groups. The area fraction f is based on a previous analysis of TEM micrographs, in which specific attention was given to control against tissue swelling artifacts that can occur during sample handling or treatment in aqueous solutions [34]. We then used this equation together the mechanical properties of the non-collagenous matrix (**σ**_M_) from tensile tests in direction transverse to the main fibril direction of equine digital flexor tendons and assuming an isotropic homogenous matrix. These revealed a negligible potential contribution of the non-collagenous matrix to axial mechanical function, since the maximal transverse stress in MGO treated samples was **σ**_M_<0.25 MPa. After excluding for these potential biases, the maximal value of the numerical gradient of the collagen fibril stress (**σ**_F_) as a function fibril deformation, D, was used to estimate collagen fibril stiffness, with units of MPa/nm, a departure from the standard definition of microstructural stiffness, e.g. µN/nm (Fig. 4 A). In conclusion, collagen fibril stiffness remained unaffected by the treatment (control: 39.9±14.7 MPa/nm; 6 h MGO: 39.7±5.6 MPa/nm; 24 h MGO: 36.2±3.9 MPa/nm; two-way ANOVA: experimental set and treatment: p = 0.611) (Fig. 4 A). This is in contrast to the tissue level mechanical properties where the elastic modulus notably increases by 35%. To provide a basis for comparison to the literature, we additionally calculated the collagen fibril modulus of all samples: 2.46±0.64 GPa.

### Collagen fibril viscoelasticity

**Figure 6:**
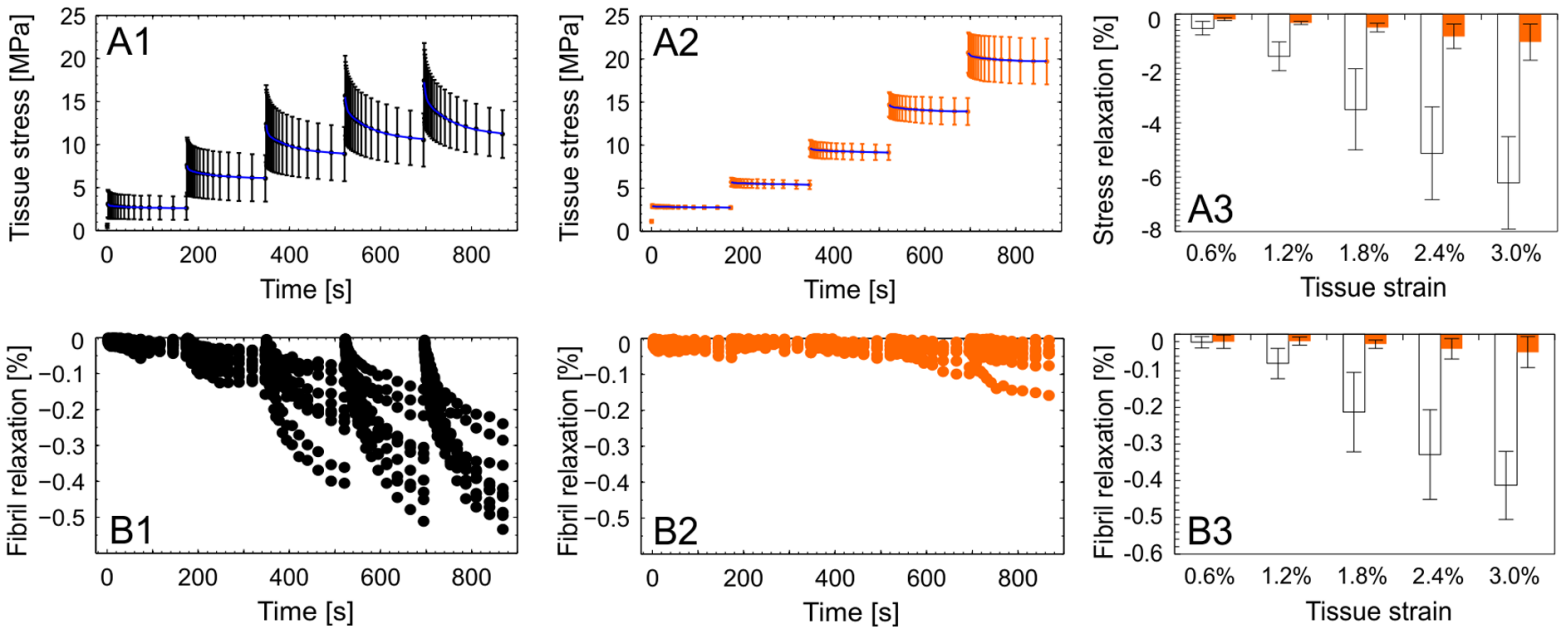
Row A) Averaged (±1 SD) tissue stress–time relations from consecutive relaxation increments of size = 0.6% L_0_: A1) Control samples (n = 10). **A2)** 6 h MGO group (n = 10). **A3)** The corresponding total stress decay. **Row B)** Collagen fibril relaxation defined as relative length change of the D-period during relaxation steps: **B1)** Controls **B2)** 6 h MGO. **B3)** The corresponding total change in fibril length.

Treatment by MGO drastically reduced collagen fibril stress relaxation. To assess collagen fibril viscoelasticity incremental stress relaxation was performed from 0% and 3% nominal strain (tissue strain, ε_T_) in increments of Δε_T_ = 0.6% (control: n = 10 and 6 h MGO: n = 10) (Fig. 6). We tested only the 6 h MGO group in combination with SAXS, since the reduction in viscoelasticity was constant beyond 6 h of treatment as mentioned before (Fig.: 3 B3). Collagen structures of rat tail tendons display two-stage relaxation kinetics at all levels of structural hierarchy [16], [35]. Thus the averaged tissue stress relaxation (**σ**_R_) and collagen fibril relaxation (**ε**_R_) were fitted by a double exponential decay for each step of relaxation: 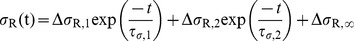
and
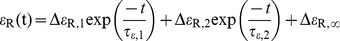



where **Δε**_R,1_, **Δε**_R,2_, **Δσ**_R,1_ and **Δσ**_R,2_ are fitted parameters and **Δε**_R,∞_ and **Δσ**_R,∞_ correspond to the values at t  =  infinity. The two term exponential fits were highly reliable for both groups in stress relaxation and for controls in fibril relaxation (R^2^>0.99) and consistently better than one term exponentials. Slightly smaller values were found for fibril relaxation of the 6 h MGO group (R^2^ = 0.78–0.99) due to small changes in relaxation compared a relatively larger variability. In conclusion, the results indicate that relaxation was drastically diminished but the characteristic two-phase behavior remained after MGO treatment when comparing values at comparable fibril elongation: For example at D≅68 nm, which corresponds to the strain increment: **ε**_T_ = 1.8% for controls and: **ε**_T_ = 3.0% for 6 h MGO, the relaxation times (**τ_σ_**, **τ_ε_**) remained very similar and only the magnitudes (**Δσ**_T_, **Δε**_F_) decreased by the MGO cross-linking (Table 1).

**Table 1:** The parameters and upper (UCI) and lower (LCI) 95% confidence intervals that were fitted to the stress and fibril relaxation at comparable fibril elongations.

	Stress relaxation			Fibril relaxation		
		Control group	6 h MGO		Control group	6 h MGO
**Fitted parameters**	**Δ???_R,1_ [Mpa]**	2.4	0.68	**Δε_R,1_ [%]**	0.16	0.032
	LCI	2.3	0.65	LCI	0.15	0.027
	UCI	2.5	0.71	UCI	0.17	0.040
	**τ_???,1_ [s]**	60	64	**τ_ε,1_ [s]**	75	68
	LCI	49.0	53	LCI	61	33
	UCI	71	76	UCI	90	99
	**Δ???_R,2_ [Mpa]**	1.1	0.37	**Δε_R,2_ [%]**	0.06	0.019
	LCI	1.0	0.33	LCI	0.05	0.014
	UCI	1.2	0.41	UCI	0.07	0.024
	**τ_???,2_ [s]**	2.8	2.9	**τ_ε,2_ [s]**	4.1	3.8
	LCI	2.0	2.3	LCI	3.1	1.5
	UCI	3.5	3.6	UCI	5.2	5.1
	**Δ???_R,∞_ [Mpa]**	8.8	19.6	**Δε_R,∞_ [s]**	0.77	0.95
	LCI	8.7	19.7	LCI	0.76	0.94
	UCI	8.9	19.6	UCI	0.78	0.96
	R^2^	0.99	0.99	R^2^	0.99	0.99

Finally, collagen fibril viscoelasticity was observed to be markedly diminished in MGO treated samples at higher levels of strain. To make this analysis we first approximated an “instantaneous” modulus (**Δσ**_b_/**Δε**_b_) at the beginning (b) of the relaxation increment. This measure was intended to capture both elastic and viscous effects, even though it is a crude approximation given the finite tissue strain-rate that was applied (0.5% **ε**_T_ s^−1^). Similarly, the elastic or “equilibrium” modulus was calculated (**Δσ**_e/_ **Δε**_e_) using values from the end (e) of the relaxation increment. This equilibrium modulus is again a crude approximation given the clearly non-infinite relaxation time of 175 s. Using these approximate measures, we observed a small and very similar viscous response in both groups at lower strains (**ε**_T_ = 0.6–1.2%), since the elastic fibril modulus was roughly 5% less than the total modulus in both groups (RM ANOVA, elastic contribution: p = 0.017, treatment group: p = 0.13). However, with increasing strains (**ε**_T_ = 1.2–1.8%) the viscous component increased in controls to approximately 10% and decreased to approximately 2% in the MGO group (RM ANOVA, elastic contribution: p = 0.024, treatment group: p = 0.072). At higher strains, post yield point, the controls began partially to fail, precluding additional analysis.

### Evidence of AGEs formed by MGO

In order to obtain insight into the type of modifications resulting from MGO, fascicle collagens were subjected to enzymatic digestion with papain to measure the acid labile fluorescence and acid hydrolysis to measure selected acid stable modifications of lysine and arginine residues.

Formation of acid labile AGEs was first confirmed by a fluorometric assay (excitation wavelength: 370 nm, emission wavelength: 440 nm) after enzymatic papain digestion and normalization against a quinine sulfate standard row *(see: Collagen fluorescence and AGE quantification in Text S1)* [26]. AGE concentration in the tissue significantly increased across all analyzed time points and followed a first order rate equation (ANOVA and all post hoc: p≤0.044) (*Fig. S1*).

**Figure 7:**
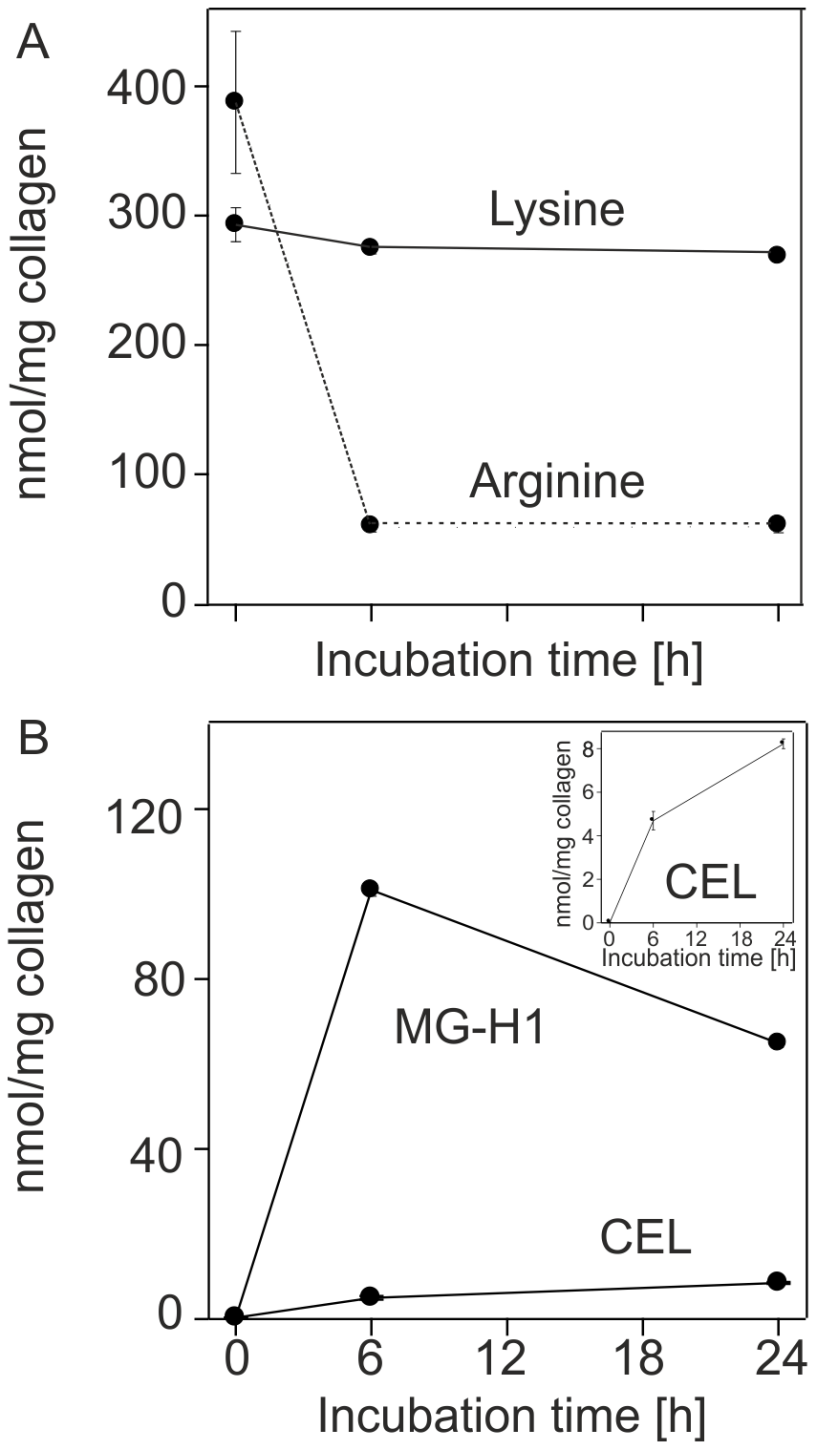
Top) The effect of 30 mM MGO on lysine and arginine content on collagen, normalized to collagen mass measured with a hydroxyproline assay and assuming 14% hydroxyproline per collagen. Averages are given with ±1 SD. **Bottom)** Effects of the MGO treatment on selected AGEs normalized by collagen content.

Analysis of arginine and lysine residue content in the acid hydrolysate was then made by LC/MS/MS using an isotope dilution technique. This analysis revealed profound losses of arginine residues (Fig. 7 A), very consistent with AGE formation. About 85% were modified by 6 h of incubation with 30 mM MGO. The MGO hydroimidazolone MG-H1, an arginine AGE, accounted for about 30% of lost arginine residues, but more likely 60% since it is partly destroyed during acid hydrolysis (Fig. 7 B). Thus, some 40% of the losses are unaccounted for, but are likely the result of degradation products of the acid labile lysine-arginine cross-link MODIC [36] and the arginine adducts N(delta)-(4-carboxy-4,6-dimethyl-5,6-dihydroxy-1,4,5,6-tetrahydropyrimidin-2-yl)ornithine (THP) as well as argpyrimidine [37]. In contrast only 10 to 15% of total lysine residues were lost from which about 50% (8 nmol) are attributable to acid stable MGO lysine adduct carboxyehtyl-lysine (CEL). The remaining portion is likely due to the lysine-lysine cross-link MOLD [38]. Among other AGEs and glycation modifications that were measured but found unchanged are furosine (fructose-lysine) and carboxyethyl-lysine (CML).

## Discussion

Collagen cross-linking by AGEs has been increasingly implicated as a central factor in the onset and progression of connective tissue disease [7]–[11]. For the first time we report the physical effects of AGEs on collagen molecular and supramolecular deformations under load. We identify and describe altered damage mechanisms that could play a central role in connective tissue disease processes. Our data provide evidence that accumulation of AGEs dramatically affects collagen fibril failure behavior and stress relaxation. These functional parameters strongly reflect how collagen structures accommodate mechanical load and overload. Because the temporal and spatial dynamics of connective tissue damage and repair involve an intricate balance of mechanically driven catabolic and anabolic processes, even slight changes in collagen mechanics or patterns of damage accumulation may detrimentally affect tissue homeostasis. Such changes in extracellular matrix mechanics are likely to be exacerbated by resistance of AGE modified substrates to proteolytic enzymes that drive and regulate balanced matrix remodeling, or by chronic activation of inflammatory mediators that drive fibrosis [39].

We selected the metabolite MGO to rapidly induce the formation of AGEs in type-I collagen fibrils on the basis that MGO chemistry is well known and that MGO induced AGE formation is both rapid and well understood [28], [36]–[38]. While *in vitro* models of AGE formation have employed a variety of buffers containing different sugars or carbonyls, these models collectively show consistent effects on both structural [40] and mechanical parameters [2], [3], [24]. It should be noted that the high MGO concentrations of up to 30 mM used in the present study clearly exceed physiological levels. Nevertheless, the mechanical changes we observed are in close agreement to the limited experimental evidence in the literature using a streptozotocin-induced diabetic model [41].

We determined an equivalent collagen fibril modulus of 2.5 GPa in both MGO modified specimens and controls - a key finding that contrasts with the current and commonly held belief that age-related modifications by AGEs increase collagen supramolecular stiffness. The modulus value of our controls is in close agreement to similar values reported by others for native collagen fibrils [18], [31], [35], [42]–[44]. Our results further agree with recent molecular modelling studies predicting an independence of fibril stiffness from enzymatic cross-links up to 30% strain if a minimum number of cross-links are in place [21], [45]. This model predicts that only at strains larger than 30% will increased LOX cross-link density lead to higher moduli and more abrupt failure modes, resulting primarily from less molecular sliding. In this sense, the stochastically formed AGE investigated in the present experimental study suggest a similar but substantially earlier mechanical onset of the effects described in the theoretical investigations of enzymatic crosslinking in tissue development. However, we were unable to measure strains as large as those simulated by the model since SAXS experiments on collagen fibrils requires that tissues must retain an intact quarter-staggered conformation to yield a signal suitable for calculating fibril strain. Nevertheless, we found that MGO modified fibrils could be elongated to a higher extent before onset of material yield: controls: ∼3.5% fibril strain vs. MGO: ∼5.7% fibril strain, as predicted by numerical models of enzymatic crosslinking. MGO modification also reduced molecular side-by-side sliding and the fibril remained in an intact quarter-staggered conformation to higher levels of tissue strains. We thus conclude that elastic deformation of the collagen molecules was promoted by MGO induced AGEs.

Another important finding was the characterization of distinctly different failure behavior in MGO treated specimens, with dramatically increased collagen fibril strength and a shift to an abrupt mode of failure. These conclusions should be considered in view of the fact that fibril failure properties could only be inferred from tissue level mechanics and SAXS based measurements of fibril strain. Nonetheless, post yield failure in MGO treated tendons was characterized by appearance of distinct subpopulations of unloaded fibrils among (still intact) mechanically loaded fibrils. This indicates that some fibrils fractured at a location somewhere along their length with the remaining length of these fibrils remaining in an intact quarter-staggered arrangement. Given that the D-period length of the fractured fibril subpopulation did not relax completely back to an unloaded state, they apparently remain slightly stretched, probably due to lateral connections to adjacent fibrils that were still under load. This parallels observations from mechanical tests within diabetic rat tissues, in which distinct failure behavior in these tissues has been demonstrated to be a consequence of increased lateral interconnectivity across multiple collagen fibrils that simultaneously fail [46]. This is in contrast to failure behavior of native samples, where signal intensities decreased after yielding without any peak splitting, indicating either a thinning of the sample or more likely the loss of quarter-staggered organization.

A third key finding is that MGO treatment reduces inherent collagen fibril viscoelasticity. Our previous work has shown that MGO treatment inhibits tendon stress relaxation [26], which is thought to be mainly governed by collagen fiber shear and sliding [16]. The results from the present study indicate that a reduced fibril viscoelasticity may also be a substantial factor in the loss of viscous response at the fiber level and the related shear matrix. However, it remains unclear how nano-scale effects of AGE could translate to mechanical effects across these different orders of magnitude in scale. An eventual involvement of AGE altered fibril surface charges [47] can in principle change the viscoelastic mechanical properties of soft tissue [48]. Comparing our observation of AGE mediated loss of fibril viscoelasticity to others, Svensson and colleagues found no mechanical differences in single fibrils from diabetic rats (Zucker diabetic fat) and that the investigated biochemical markers of AGE remained equal to controls [42].

Considering what is known from the biomaterials field regarding chemical cross-linking of collagen constructs, Yang and colleagues have described distinct effects on single collagen fibril viscoelasticity using cross-linking reagents of different cross-linking length [35]. With the “zero length” cross-linker EDC (1-Ethyl-3-(3-dimethylaminopropyl)-carbodiimide) there was no change of fibril modulus found aside from an increased failure behavior and a reduced viscoelasticity, which echoes the results of the present study (*Fig. S5*). In contrast, glutaraldehyde formed cross-links have a length of least 1.3 nm length [35], [49] that can bridge across micro-fibrils, and glutaraldehyde treatment has been shown to increase fibril modulus [35]. Based on these observation we hypothesize that the lack of an increased fibril modulus in MGO cross-linked tendon is due to its shorter cross-linking length in the range from 0.26 nm to 0.70 nm [22], [50].

There are some potential weaknesses of this study. First, despite clear increases in fluorescence and denaturation temperature [26] after the applied treatment we have not provided direct molecular evidence for cross-linking by MGO, although extensive MOLD and MODIC formation has been documented in the course of protein incubation by methylglyoxal [51]. Secondly, while we suspect that the fibril shell is more susceptible to AGE modification than the fibril interior [52], the data we present preclude any meaningful analysis of AGE location and conjecture regarding where they form on the basis of our data would be speculative at best. Thirdly, concerning the physiological relevance of the employed *in vitro* AGE model, the high MGO concentrations used in this study were decidedly non-physiological and are to some extent analogous to the use of glutaraldehyde as a fixative agent. Finally, we cannot exclude the possibility that acute cross-linking by MGO could non-physiologically alter multi-scale shear transfer mechanisms in the non-collagen matrix. Although, our experiments investigating the load bearing capacity of the non-collagen matrix indicated that contribution of these AGE modified matrix components (mechanical properties when the tendon is tested transverse to the functional axis of the tissue) to tissue strength was minimal. *In vivo*, the cells and non-collagen matrix would be less susceptible to AGE accumulation given their relatively short half-life in comparison to type-I collagen [13]. We nonetheless accept these limitations that accompany the employed *in vitro* AGE model, which avoids critically confounding effects (i.e. collagen loss) in comparative models of endogenous AGE formation.

Generally, our use of the D-period length as a proxy measure of fibril deformation and estimates of gap and overlap length agree well with reported values from previous SAXS experiments [14], [15], [18], [19], [53]. Fibril strains were always less than the applied tissue strains. The ratio of fibril to applied strain was 0.6, which is considerably higher compared to previously reported values of 0.4 [19], [54]. We attribute this difference to the at least 5-fold higher strain-rate used in the present work compared to previous studies, which minimized viscoelastic effects over the time course of the experiment. More interestingly however was the fact that this ratio increased to 0.75 when the applied tissue strain (based on grip-to-grip displacements) was corrected to an estimate of mid-substance strain *(for the relation of grip-to-grip tissue strain versus tissues strain based on optical markers see: Correction for local-optical strain measurements in Text S1)* that corrects for deformation “artifacts” at the clamps. We also note the similarity of our collagen fibril relaxation data to Gupta and colleagues [16]. This is particularly interesting, since their results come from a single relaxation experiment per sample, in contrast to the present work in which each sample underwent multiple relaxations across a range of strain levels. Thus collagen fibril relaxation seems to be at least partly independent of strain history.

In conclusion, our data reveal that MGO-induced AGEs reduce inherent collagen fibril viscoelasticity and alter fibril failure mode. We attribute these changes in viscous response and failure to altered molecular sliding as measured in the present study. We also conclude that tensile stiffness (modulus) changes in tissue are not due to an increased stiffness at the fibril level, and conclude that this must largely be due to changes in higher collagen structures [26]. Collectively these findings add considerable precision to the very general clinical observation that connective tissue mechanical properties change with age and diabetes. These findings also sharpen focus on the range of possible vectors by which AGE accumulation in collagen tissue is likely to affect tissue homeostasis and drive tissue pathology.

## Experimental Procedures

### Sample preparation and AGE induction

Tails from skeletally mature Sprague-Dawley rats (17 to 24 weeks) were removed after sacrifice and stored (−18°C) until the day of experiment, which should not have an effect on tendon mechanics [55]. Rats have been euthanized by carbon dioxide inhalation for an unrelated study. Rats were handled in strict accordance with Swiss regulations for animal studies according to the Federal Food Safety and Veterinary Office. The study was previously approved by the ethics committee of the Canton Zurich Veterinary Office. For each set of experiment the age of the animals was matched. Individual fascicles were dissected and mounted in poly-sulfone clamps followed by area measurements at a pre-stress of 1.5 MPa (details follow). AGEs were induced by incubation at 36°C with 5 ml of 30 mM MGO *(see: Synthesis of high quality methylglyoxal in Text S1)* buffered with 100 mM EPPS (4-(2-Hydrox-yethyl)-1-Piperazinepropanesulfonic acid) in PBS (pH = 8) containing protease inhibitors (1 mM EDTA, 2 mM AEBSF, 130 µM betastatin, 14 µM E-64, 1 µM leupeptin and 0.3 µM aproptin). Mechanical tests after treatment with MGO in phosphate buffered saline (PBS, pH = 7.4) indicated equal effects. Finally, the reaction of MGO was ceased by multiple washing steps in copious amounts of PBS on ice for at least 2 h. The specificity of the treatment effects was investigated by incubations in presence of L-arginine (20 mM) and L-lysine (20 mM). A fluorometric assay was used to measure collagen associated fluorescence *(see: Collagen fluorescence and AGE quantification in Text S1)* [24].

### Determination of advanced glycation end-products

After treatment tendons were washed with water and extracted with 2:

1 chloroform-methanol for 24 h followed by rehydration with water and freeze-dried. Approximately 1 mg of each sample was acid hydrolyzed in 1 ml of 6 M HCl under nitrogen for 16 h at 110°C. The acid was evaporated by a speed-vac concentrator (Thermo-Fisher) followed by reconstitution of the material in 0.5 ml water and filtered (0.45 µm filter, Spin-X, Corning Costar). The collagen content was determined by the hydroxyproline assay [56]. Aliquots of 100 µg collagen were spiked with isotopocially labeled internal standards and analyzed by injection of 40 µl (∼40 µg) into a high-performance liquid chromatography - mass spectrometer (HPLC-MS/MS) for lysine, arginine, MGO hydroimidazolone (MG-H1) and carboxyethyl-lysine (CEL) as previously described [57].

### Biophysical characterization of the AGE-tendon model

Tendon fascicles were quasi-statically tested in uniaxial tension in a custom climate chamber containing PBS placed on a universal testing machine (Zwick, Z010 TN, 20 N load-cell, Germany). The initial length of the sample (L_0_) was measured from clamp-to-clamp at a pre-stress of 1.5 MPa (*Fig. S2*). At this point, two digital photographic images from orthogonal perspectives were taken (Telecentric lenses: Carl Zeiss, Visionmes 16/1/1, Germany; Cameras: iDS, UI-5250CP, pixel size: 4.5 µm, Germany) to characterize the area (minor diameters of the ellipsoid shaped fascicle ≥180 µm)[34], [55]. Tissue strain was defined as nominal strain: ε_T_ = (L – L_0_)/L_0_ [%] *(for a comparison to optical markers derived strains see: Correction for local-optical strain measurements in Text S1)*. Before all mechanical tests, tendons were preconditioned up to 1.5% L_0_ until stress-strain curves were congruent. Subsequently, relaxation or ramp to failure experiment was conducted with constant strain-rates (0.5% L_0_ s^−1^). The force readings were normalized to (nominal) tissue stress (σ_T_) by the pre-treatment sample area. Normalized stress relaxation was defined as σ_R_(t) = 1- σ(t)/σ(0) [%], with σ(0) being the stress at start of each relaxation step and the time (t) ranging from 0 to 175 s. The tissue mechanical properties were described by elastic modulus in the linear range of the stress-strain relation using linear regression. Onset of plastic deformation, yield point, was estimated using a 0.2% L_0_ offset of the regression line in the linear part of the stress-strain curve. Tissue failure was characterized by ultimate strength (UTS) and the corresponding strain [58]–[60]. MGO significantly shortened the sample length at pre-stress of 1.5 MPa by 3% (ANOVA: p = 0.049), leading to a slight corresponding underestimation of elastic modulus in cross-linked samples by approximately 3%. This bias was considered to be negligible compared to the observed effects and was therefore not considered.

### Synchrotron X-ray scattering measurements

Frozen tails were stored on dry ice for transportation to the beamline facilities and then stored at −18°C until testing. Preliminary experiments indicated no changes in the mechanical properties of the tissue. Following sample preparation, as described before, fascicle-clamp assemblies were mounted on a custom-built tensile testing apparatus (linear stepper motor: NA23C60, accuracy 40 µm, Zaber Technologies Inc., Canada; load cell: KD24S, 20 N, accuracy: 0.1%, Transmetra, Switzerland) with a custom-designed control software (LabVIEW). An x-y table was used to adjust the axial sample alignment. The tensile tester was a modification of an available micro-compression device [61], which was designed to fit on the stage of the coherent small-angle X-ray scattering (cSAXS) beamline at Swiss Light Source (Paul Scherrer Institute, Switzerland). During all experiments two 30 µm Kapton films (X-ray semitransparent material) were placed on two sides of each tendon held together by a drop of PBS to maintain tissue hydration (*Fig. S3*)[16].

To measure collagen fibril deformation we used SAXS with an X-ray wavelength of 0.1 nm obtained from a double-crystal Si(111) monochromator and a beam cross-section at the sample 300 µm width and 200 µm height obtained by slight focusing using the bendable monochromator crystal and high-order rejection mirror. The specimens were placed ∼12 mm upstream the entrance window of an evacuated 7.03 m flight tube. 2D diffraction patterns were recorded with PSI developed PILATUS 2M detector (array size: 1475×1679 pixels; pixel size: 172×172 µm2; active area: 254×289 mm^2^) [62]. The total sample to detector distance (7.158 m) and the center of the direct beam on the detector were calibrated using silver behenate. The SAXS frames were collected with an acquisition time of 200 ms concurrently during mechanical testing with the detector being triggered by the testing apparatus. During each relaxation step (subsequent tissue strain increments of **Δε**_T_ = 0.6% L_0_) frames were acquired at exponentially increasing time intervals. This exponential curve matched previous published collagen fibril relaxation by Gupta [16] and allowed an optimal acquisition of data where collagen deformation occurred. A shutter closing between frames reduced the X-ray dose at the sample *(for the effect of X-ray irradiation on the experimental measures see Fig. S4)*.

### SAXS data analysis

The collected 2D SAXS patterns showed the 1^st^ to 12^th^ order meridional collagen reflections. Azimuthal integration was carried out over angular sectors of 45° width centered at the calibrated location of the direct beam on the detector. From the resulting 1D intensity profiles [a.u.] versus q [nm^−1^] (Fig. 5 A) a two-term exponential background was subtracted (empirically determined best fit). The peak position, height and width (FWHM) were estimated by fitting a series of Gaussian curves to the first 8 order collagen reflections using non-linear least squares (OriginPro 8.6; Matlab R2013a). A straight line, q(n) = a+b * n, was fitted through the peak positions q(n) as a function of the order number (n) [16]. The collagen fibril D-spacing was calculated from D = 2 π/b. The precision of measuring the D-period was 0.24%. Precision was measured at pre-stress and is given as the upper 95% confidence interval of the coefficient of variation from 26 samples. Either absolute or normalized change of D-spacing served as averaged measures of collagen fibril elongation: collagen fibril deformation/lengthening (D) and collagen fibril strain (**ε**_F_ = D/D_0_–1 [%]) with D_0_ as D-period length at pre-stress/start of experiment. For each relaxation step, collagen fibril relaxation was measured as the relative shortening of D normalized to the D at start of each relaxation step (D_0_): **ε**_R_ = D/D_0_–1 [%]. Collagen fibril deformation as a function of tissue strain was highly linear up to at least yield point (Fig. 5 C) and therefore fitted by 1^st^ order polynomials to calculate fibril stiffness. Force measurements were smoothed using a moving average filter of 20 points to reduce noise of the data recorded at 100 Hz.

### Statistical analysis

Parameters derived from the experiments were analyzed using t-tests (factor: treatment group), one-way ANOVAs (fixed factor: treatment group), two-way ANOVAs (fixed factor: treatment group and experimental set) or RM ANOVAs (fixed factor: treatment group, repeated measures factor: time of treatment). Post hoc pairwise comparisons were made with adjusted p-values (Dunnett's test for treatment vs. control, Tukey-Kramer test for all pairwise comparisons with unequal sample sizes). Unless otherwise stated, results are reported as averages and standard deviations (1 SD). Two-sided tests were used and p-values of 0.05 were chosen as the level of significance, if not stated otherwise. Corresponding non-parametric counterparts were used when model assumptions were not met (SPSS v21.0/Matlab R2013a/OriginPro 8.6).

## Supporting Information

Figure S1 **Results from collagen fluorescence and AGE fluorometric measurements.** Accumulation of AGEs in tendon specimens after 0 h, 6 h, 24 h and 96 h of incubation in MGO. Each data point, respectively sample, was measured thrice and averaged (relative precision: coefficient of variation  = 7%).
(TIF)

Figure S2 **A) A measurement of grip-to-grip/machine strain vs. mid-substance tissue strains.** The relationship was highly linear and could therefore be well approximated by a line (least square fitting) and reduced to one measure (ratio: εO/εT) that could be used to correct all measurements. **B)** A tendon-clamp assembly in the fixation rig. The white paper was used to increase the friction at the clamps. Before each test dark markers were gently applied for optical strain measurements.
(TIF)

Figure S3 **Consecutive force-strain cycles from one sample out of 5, that were used to control for any biases on sample mechanics when using the Kapton mini-climate chamber (either due to evaporation of the PBS and corresponding sample dehydration or due to directly affecting the force reading).** To clearly separate the effect of dehydration from normal sample mechanics, the sample was also tested after drying for 30 min on air.
(TIF)

Figure S4 **Effects of X-ray irradiation (12.4 keV) of one exemplar sample.** The gray area indicates the maximal irradiation time that a sample was exposed to during any mechanical experiments. A) The calculated D-period lengths are shown for the first eight order collagen reflections. The higher the D-period value, the lower the order of the reflection. One can observe the systematic error of measuring the D-period by the slight differences in values from different orders, which is a result probably of a slight asymmetry in the lower order peaks, mainly 1 and 2. **B)** Shown are the intensities from these different ordered reflections. These radiation effects were irreversible, unless we repeated the experiments at a different spot on the tendon.
(TIF)

Figure S5 **Normalized stress relaxation with σR(t) as the stress at time (t) and σR(0) the stress at start of the relaxation.** Control and 6 h MGO are stress relaxation experiments at similar collagen deformation levels (68 nm) and are taken from the present study. For comparison values from single collagen fibril relaxation are taken from Yang et al. [35]. They also fitted their data with two exponentials. The fitted parameters were taken and plotted here (green).
(TIF)

Text S1 **Supporting Information Methods.**
(DOCX)
