# Supplementary figures and images for: Advanced Glycation End-Products Reduce Collagen Molecular Sliding to Affect Collagen Fibril Damage Mechanisms but Not Stiffness
Source: PLoS One. 2014 Nov 3;9(11):e110948. doi: 10.1371/journal.pone.0110948 (PMC4217736; doi:10.1371/journal.pone.0110948)

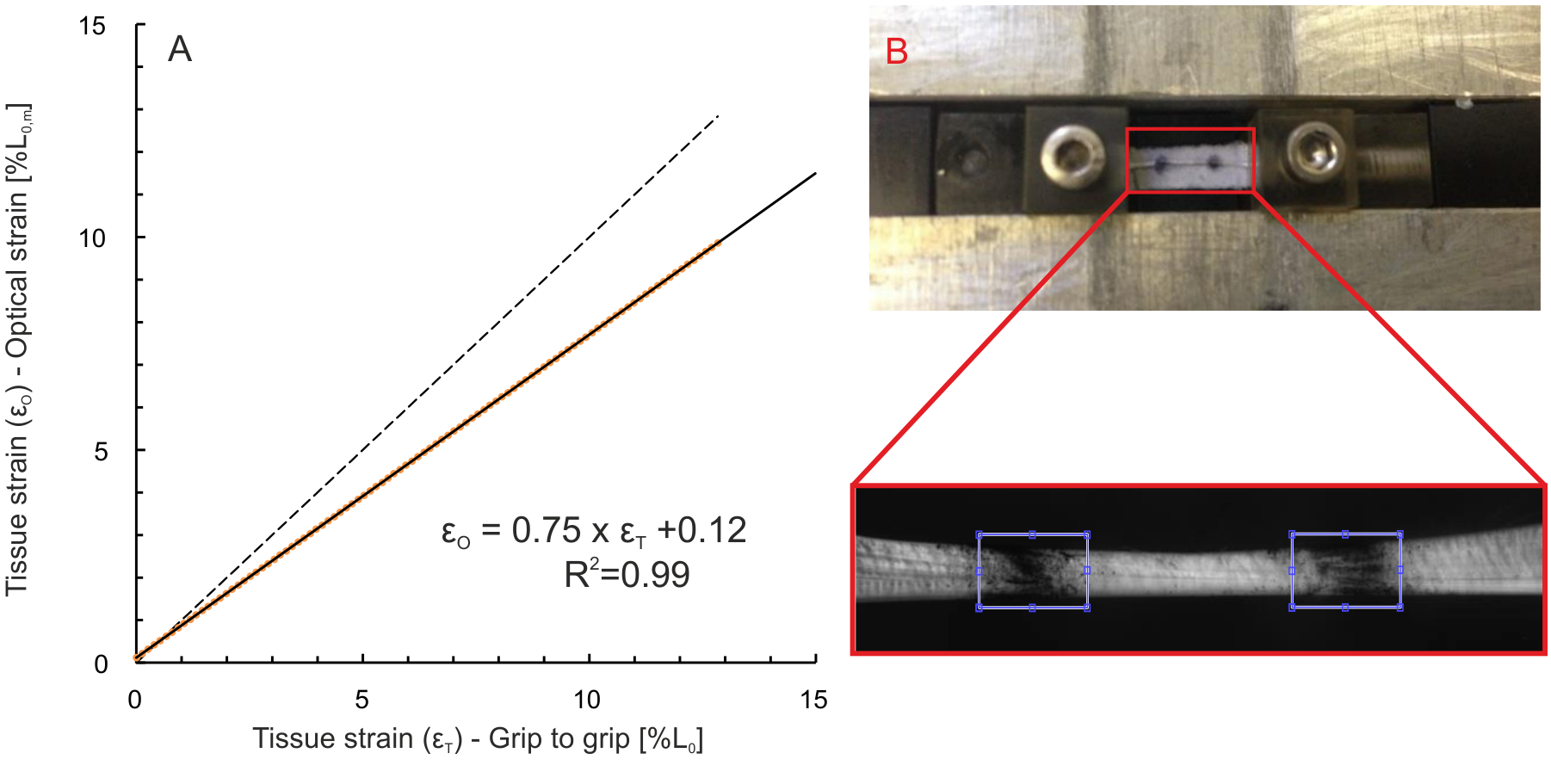

Supplement: Figure S1 — Results from collagen fluorescence and AGE fluorometric measurements. Accumulation of AGEs in tendon specimens after 0 h, 6 h, 24 h and 96 h of incubation in MGO. Each data point, respectively sample, was measured thrice and averaged (relative precision: coefficient of variation = 7%). (TIF) [file pone.0110948.s001.tif]

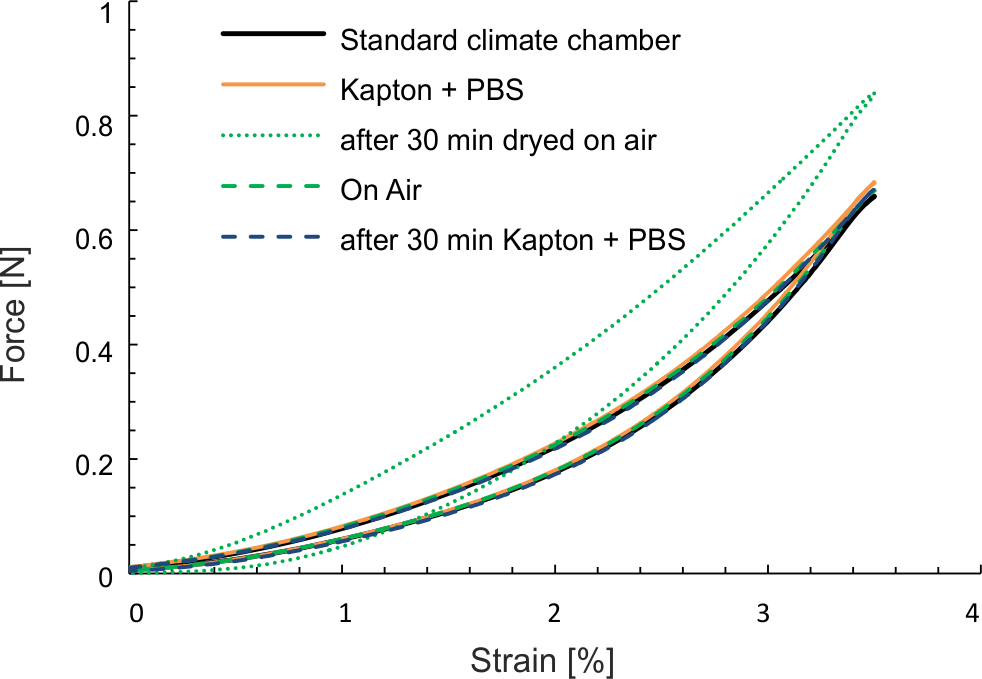

Supplement: Figure S2 — A) A measurement of grip-to-grip/machine strain vs. mid-substance tissue strains. The relationship was highly linear and could therefore be well approximated by a line (least square fitting) and reduced to one measure (ratio: εO/εT) that could be used to correct all measurements. B) A tendon-clamp assembly in the fixation rig. The white paper was used to increase the friction at the clamps. Before each test dark markers were gently applied for optical strain measurements. (TIF) [file pone.0110948.s002.tif]

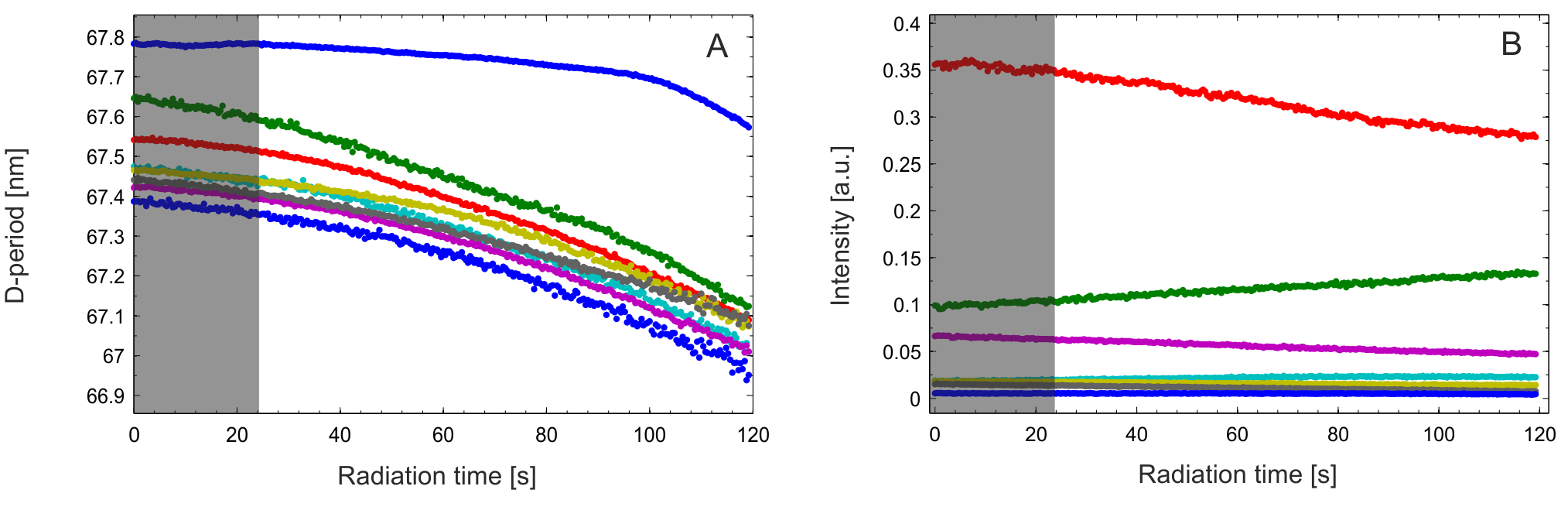

Supplement: Figure S3 — Consecutive force-strain cycles from one sample out of 5, that were used to control for any biases on sample mechanics when using the Kapton mini-climate chamber (either due to evaporation of the PBS and corresponding sample dehydration or due to directly affecting the force reading). To clearly separate the effect of dehydration from normal sample mechanics, the sample was also tested after drying for 30 min on air. (TIF) [file pone.0110948.s003.tif]

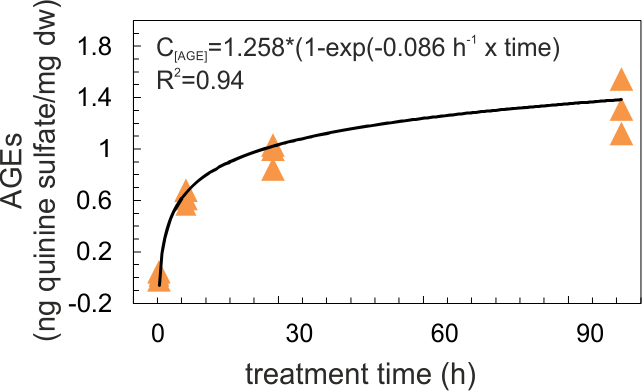

Supplement: Figure S4 — Effects of X-ray irradiation (12.4 keV) of one exemplar sample. The gray area indicates the maximal irradiation time that a sample was exposed to during any mechanical experiments. A) The calculated D-period lengths are shown for the first eight order collagen reflections. The higher the D-period value, the lower the order of the reflection. One can observe the systematic error of measuring the D-period by the slight differences in values from different orders, which is a result probably of a slight asymmetry in the lower order peaks, mainly 1 and 2. B) Shown are the intensities from these different ordered reflections. These radiation effects were irreversible, unless we repeated the experiments at a different spot on the tendon. (TIF) [file pone.0110948.s004.tif]

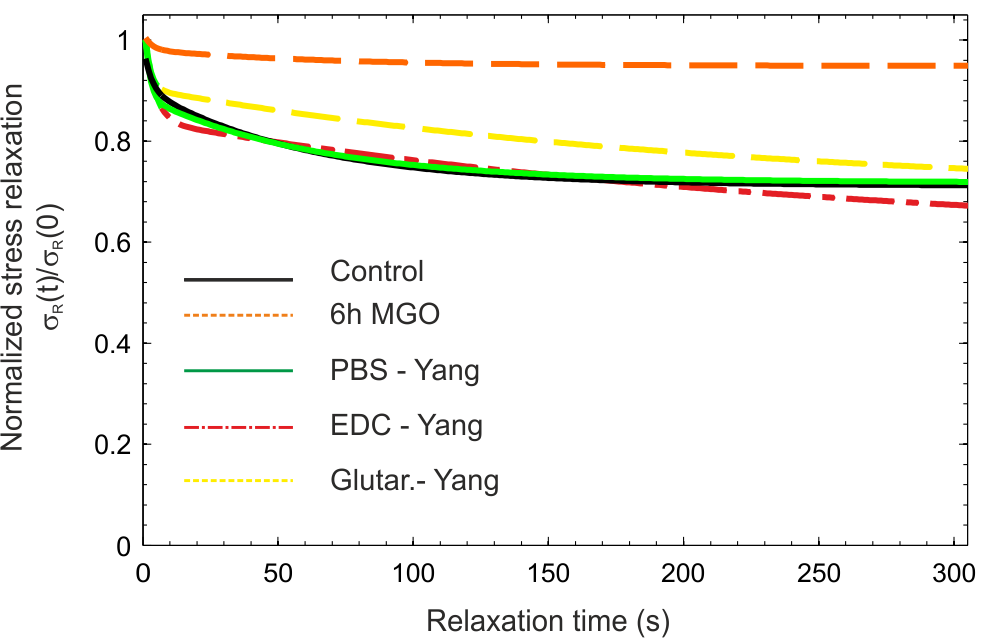

Supplement: Figure S5 — Normalized stress relaxation with σR(t) as the stress at time (t) and σR(0) the stress at start of the relaxation. Control and 6 h MGO are stress relaxation experiments at similar collagen deformation levels (68 nm) and are taken from the present study. For comparison values from single collagen fibril relaxation are taken from Yang et al. [35]. They also fitted their data with two exponentials. The fitted parameters were taken and plotted here (green). (TIF) [file pone.0110948.s005.tif]
