# Supplementary material for: Advanced Glycation End-Products Reduce Collagen Molecular Sliding to Affect Collagen Fibril Damage Mechanisms but Not Stiffness
Source: PLoS One. 2014 Nov 3;9(11):e110948. doi: 10.1371/journal.pone.0110948 (PMC4217736; doi:10.1371/journal.pone.0110948)
Supplement: Text S1 — Supporting Information Methods. (DOCX) [file pone.0110948.s006.docx]

**SUPPORTING INFORMATION METHODS**

**Synthesis of high quality methylglyoxal**

Highly purified methylglyoxal (MGO) was synthesized [[1](#_ENREF_1)] to remove potential contaminants in commercial MGO solution [[2](#_ENREF_2), [3](#_ENREF_3)]. In brief, pyruvic aldehyde dimethyl acetal (41.31 mmol, Aldrich 170216, ≥ 97 %) was hydrolyzed in 100 mL sulfuric acid (5 %, v/v) over a boiling water bath to form MGO. Fractional distillation under reduced pressure (30 mbar, boiling point of the azeotrop = 26 °C) and nitrogen bleed removed methanol and unreacted pyruvic aldehyde dimethyl acetal before the product was recovered. In a light protected environment, MGO was derivatized with 1,2-phenyldiamine (Aldrich P23938, 99.5%) to 2-methylquinoxaline. Separation and quantitative analysis of the derivatization product was done through HPLC with a mixed-mode column from Zeochem (Uetikon am See, Switzerland) and a UV detector at 229 nm and 325 nm, comparing against 2-methylquinoxaline (Aldrich W511609, ≥97%) standards [4]. Sample quality was verified with ^1^H-NMR spectroscopy (in (D2O)[5]. Multiple repetitions yielded highly purified MGO solutions of at least 0.6 M with negligible methanol contaminations and MGO mono- (42 %) to di-hydrate (58 %) ratios as documented [1-5].

**Collagen fluorescence and AGE quantification**

Additional tail tendons (~ 20 mg) were washed and cross-linked directly, washed in buffer, centrifuge dried under vacuum for 36 h and weight. The total AGE content was approximated using a modified fluorometric assay [[4](#_ENREF_4)]. Samples were digested with papain (P4762 Sigma, ~ 0.05 unit enzyme / mg tissue in 0.1 M Na_2_HPO_4_ and 0.01 M Na_2_EDTA, pH = 6) for 18 h at 65 °C. Triplicate aliquots were then assayed for fluorescence (excitation wavelength: 370 nm, emission wavelength: 440 nm) against a quinine sulfate standard row (Q1250 Sigma, ≥ 90%) using a microplate spectrofluorometer (SPECTRAmax GEMINI XS, Molecular Devices, USA). For results see Fig. S1.

**Non-collagenous matrix characterization**

The transverse mechanical properties of tendon were used to estimate a potential contribution/bias of the treatment by affecting the non-collagenous matrix (cross-linked glycoproteins, proteoglycans or cells) that could contribute to the found observations. Due to the small size of rat tail tendons, we used equine superficial digital flexor tendons instead. Further, equine flexor tendons are composed of well aligned collagen fibrils. Equine flexor tendons were cut transversely to the main resident collagen fiber-direction using a hand-held custom device fitted with three parallel microtome blades (high profile, PTFE coated, 7310, Dura Edge)[[5](#_ENREF_5)]. This yielded ~30mm long transverse tendon strips free from surrounding connective tissue (epitenon) with an averaged cross-sectional area of 3.6 ± 0.3mm^2^ (electronic caliper: accuracy of 0.05 mm). After cross-linking the samples were glued with cyanoacrylate between two pieces of sandpaper that served as clamps. A stochastic graphite surface pattern was applied to facilitate video extensometry at 15 fps (Telecentric lens: VS-TC0510, VS Technology Corporation, Japan). **SIFT feature extraction [**[**6**](#_ENREF_6)**] was used prior to elastic image registration of c**onsecutive frames **using imageJ plug-in bUnwarpJ [**[**7**](#_ENREF_7)**]** to calculate (infinitesimal) strain fields as previously described [[5](#_ENREF_5)]. The homogenous local axial (ε_T,_*_axial_*) and transverse strains (ε_T,_*_trans_* ) were averaged over the mid-substance of the sample and used to derive tangential elastic modulus and poisons ratio (ν=- ε_T,_*_trans_* / ε_T,_*_axial_*).

The averaged transverse elastic modulus doubled approximately (100% increase) due to MGO treatment (control: 1.0 ± 0.77 MPa vs. MGO: 2.3 ± 1.7 MPa, t-test: p = 0.015). Using video extensometry, an averaged Poisson’s ratio of both groups showed significantly deviation from isotropy (control: 0.75 ± 0.14, t-test: p = 0.01; MGO: 0.83 ± 0.20, p < 0.001). Stress-strain curves of MGO further indicated also a more brittle material, but both being very similar to an elastic-perfect plastic material.

**Correction for local-optical strain measurements**

Rat tail tendon fascicles (n = 5) were prepared as for all other mechanical tests. Two fine marks were placed on the fascicle using an indelible marker (Eding 400, Germany) by gently tapping on the surface of the sample that is supported by a clamping rig (Fig. S2 B). Samples were then monitored by the video system used for cross-sectional measurements (at 3.75 fps). Two rectangular region of interest (ROI) were manually selected covering the marker spots including some adhering unmarked tendon tissue. Custom software using normalized 2-D cross-correlation was then used to track the ROI centroids (Matlab). Raw marker displacements were fitted by approximation to 2^nd^ order polynomials. The absolute axial marker distance (L_m_) was normalized to engineering strain based on initial marker distance (L_0,m_). Based on the highly linear relationship of machine strains and optical strains (R^2^ ≥ 0.99) the ratio of ε_O_ / ε_T_ could be calculated using the slope of least square fitted straight lines to the data (Fig. S2 A). On average the optical strains measured in the mid-substance were 80.0 ± 15 % (1SD) of the applied machine strains.

**Supplementary references:**

**1. McLellan, A.C. and P.J. Thornalley, *Synthesis and chromatography of 1,2-diamino-4,5-dimethoxybenzene, 6,7-dimethoxy-2-methylquinoxaline and 6,7-dimethoxy-2,3-dimethylquinoxaline for use in a liquid chromatographic fluorimetric assay of methylglyoxal.* Analytica Chimica Acta, 1992. 263(1–2): p. 137-142.**

**2. Pourmotabbed, T. and D.J. Creighton, *Substrate specificity of bovine liver formaldehyde dehydrogenase.* Journal of Biological Chemistry, 1986. 261(30): p. 14240-14244.**

**3. Rae, C., et al., *Kinetic analysis of the human erythrocyte glyoxalase system using 1H NMR and a computer model.* European Journal of Biochemistry, 1990. 193(1): p. 83-90.**

**4. Vashishth, D., et al., *Influence of nonenzymatic glycation on biomechanical properties of cortical bone.* Bone, 2001. 28(2): p. 195-201.**

**5. Fessel, G., et al., *Exogenous collagen cross-linking recovers tendon functional integrity in an experimental model of partial tear.* J Orthop Res, 2012. 30(6): p. 973-81.**

**6. Lowe, D.G., *Distinctive Image Features from Scale-Invariant Keypoints.* International Journal of Computer Vision, 2004. 60(2): p. 91-110.**

**7. Arganda-Carreras, I., et al., *Consistent and Elastic Registration of Histological Sections Using Vector-Spline Regularization*, in *Computer Vision Approaches to Medical Image Analysis*, R. Beichel and M. Sonka, Editors. 2006, Springer Berlin / Heidelberg. p. 85-95.**
